# Usable Mobile App for Community Education on Colorectal Cancer: Development Process and Usability Study

**DOI:** 10.2196/12103

**Published:** 2019-04-16

**Authors:** Muhamad Fadhil Mohamad Marzuki, Nor Azwany Yaacob, Najib Majdi bin Yaacob, Muhammad Radzi Abu Hassan, Shahrul Bariyah Ahmad

**Affiliations:** 1 Department of Community Medicine School of Medical Sciences Universiti Sains Malaysia Kota Bharu Malaysia; 2 Unit Biostatistics and Research Methodology School of Medical Sciences Universiti Sains Malaysia Kota Bharu Malaysia; 3 Clinical Research Centre Hospital Sultanah Bahiyah Alor Setar Malaysia; 4 Non-Communicable Disease Unit Kedah State Health Department Alor Setar Malaysia

**Keywords:** colorectal cancer, mobile app, development, mHealth

## Abstract

**Background:**

Participation in colorectal cancer screening is still low among Malaysians despite the increasing trend of incidence, with more than half of the new cases being detected in the advanced stages. Knowledge improvement might increase screening participation and thus improve the chances of disease detection. With the advancement of communication technology, people nowadays prefer to read from their mobile phone using a Web browser or mobile apps compared with the traditional printed material. Therefore, health education and promotion should adapt this behavior change in educating the community.

**Objective:**

This study aimed to document the process of designing and developing a mobile app for community education on colorectal cancer and assess the usability of the prototype.

**Methods:**

The nominal group technique (NGT) was used for the content development of the mobile app. NGT involving community educationists and clinicians combined with community representatives as the target users identified relevant health information and communication strategies including features for a user-friendly mobile app. The prototype was developed using framework Ionic 1, based on the Apache Cordova and Angular JS (Google). It was published in the Google Play store. In total, 50 mobile phone users aged 50 years and above and who had never been diagnosed with any type of cancer were invited to download and use the app. They were asked to assess the usability of the app using the validated Malay version of System Usability Scale Questionnaire for the Assessment of Mobile Apps questionnaire. The One-sample *t* test was used to assess the usability score with a cut-off value of 68 for the usable mobile app.

**Results:**

The Colorectal Cancer Awareness Application (*ColorApp*) was successfully developed in the local Malay language. The NGT discussion had suggested 6 main menus in the *ColorApp* prototype, which are Introduction, Sign and Symptoms, Risk Factors, Preventive Measures, Colorectal Cancer Screening Program, and immunochemical fecal occult blood test kit. A total of 2 additional artificial intelligence properties menus were added to allow user-*ColorApp* interaction: Analyze Your Status and *ColorApp* Calculator. The prototype has been published in the Google Play store. The mean usability score was 72 (SD 11.52), which indicates that *ColorApp* is a usable mobile app, and it can be used as a tool for community education on colorectal cancer.

**Conclusions:**

ColorApp mobile app can be used as a user-friendly tool for community education on colorectal cancer.

## Introduction

Colorectal cancer has become a prominent health problem in the twenty-first century worldwide [1]. It is the third commonest cancer and the fourth leading cause of cancer-related deaths in the world. Its burden is expected to increase by 60% to more than 2.2 million new cases and 1.1 million cancer deaths by 2030 [2]. Malaysia had reported colorectal cancer as the commonest cancer in male and the second commonest cancer in female after breast cancer with an age-standardized rate of 14.6 and 11.1 per 100,000 population, respectively, in 2016 [2].

Colorectal cancer is one of the preventable diseases that are treatable with early detection and treatment. The 5-year survival rate is highly dependent on the stage at diagnosis. Its survival ranges from 95% if detected at stage 1 to 8% if detected at stage IV [3]. In Malaysia, 65% of colorectal cancer was detected at stages III and IV [4], giving rise to lower 5-year relative survival by stage as compared with other developed Asian countries [5]. The late detection might be partly because of the low participation in screening among Malaysians; hence, this called for a more effective strategy to improve in disease knowledge [3,4].

Health education and promotion in Malaysia had utilized a variety of communication strategies including health talk and forums as well as printed material such as pamphlets and health notices. Websites on healthy lifestyles had been created with interactive self-risk-assessment features. However, these might be able to reach those who seek health information either from community health activities or online. With the increasing number of registered mobile phone users, including people in the higher age group in Malaysia [6,7], health outreach thus needs a new strategy. The Interim Review of Malaysian Citizens Reading 2014 had reported that people nowadays prefer to read from their mobile phone using a mobile app or Web browser as compared with printed material [8]. Access to information through mobile phone also seems to be more convenient than attending community activities. Thus, this study encroaches a new strategy in line with the current community behavior by developing a mobile app for a disease of public health importance. This paper aimed to document the process of designing and developing a mobile app for community education on colorectal cancer and report its usability assessment.

## Methods

The development of the mobile app prototype was conducted from November 2017 to February 2018. The developmental processes include content development, prototype development, and prototype usability assessment by the intended user. The details of the processes are provided below.

### Content Development

The content of the mobile app prototype was developed based on the theory of Health Belief Model (HBM). Content development of the prototype involved a few stages. The first stage was conducting a literature review on colorectal cancer, sign and symptoms, associated factors, screening and prevention, and the features of the mobile app for health promotion and education. This evidence-based information served as important guiding points for the next stage in identifying content relevant to the intended users. For example, participants may not be familiar with the features of a usable mobile app and they may face difficulties in giving their opinion about the features of a user-friendly app. Therefore, researchers provided information on the expected features of a mobile app by the user from published researches.

Nominal group technique (NGT) was applied to explore the following 3 elements:

1. What is the information needed by the intended users?
2. How to ensure that the information is self-explanatory?
3. What are the features needed to make the mobile app a user-friendly app?

NGT is a structured meeting that aims to provide an orderly procedure for obtaining qualitative information from target groups who are most closely related to the problem area [9]. It is a variation of small group discussion to reach a consensus. NGT gathers information by asking participants to respond to questions posed by a moderator (researcher) and then asking participants to prioritize the ideas or suggestions of all the group members [10]. This technique was developed by Delbecq and Van de Ven and comprises 4 key stages: silent generation, round robin, clarification, and voting (ranking or rating) [11].

The NGT participants consist of the following:

1. one public health physician
2. one gastroenterologist
3. one family medicine physician
4. two medical officers
5. one Assistant Environmental Health Officer (Noncommunicable Disease Control Unit)
6. three individuals from intended users
7. two researchers as moderator

Participants were contacted by phone 2 weeks before the session. They were briefed on the topic and what is the expected outcome from the discussion.

**Figure 1:**
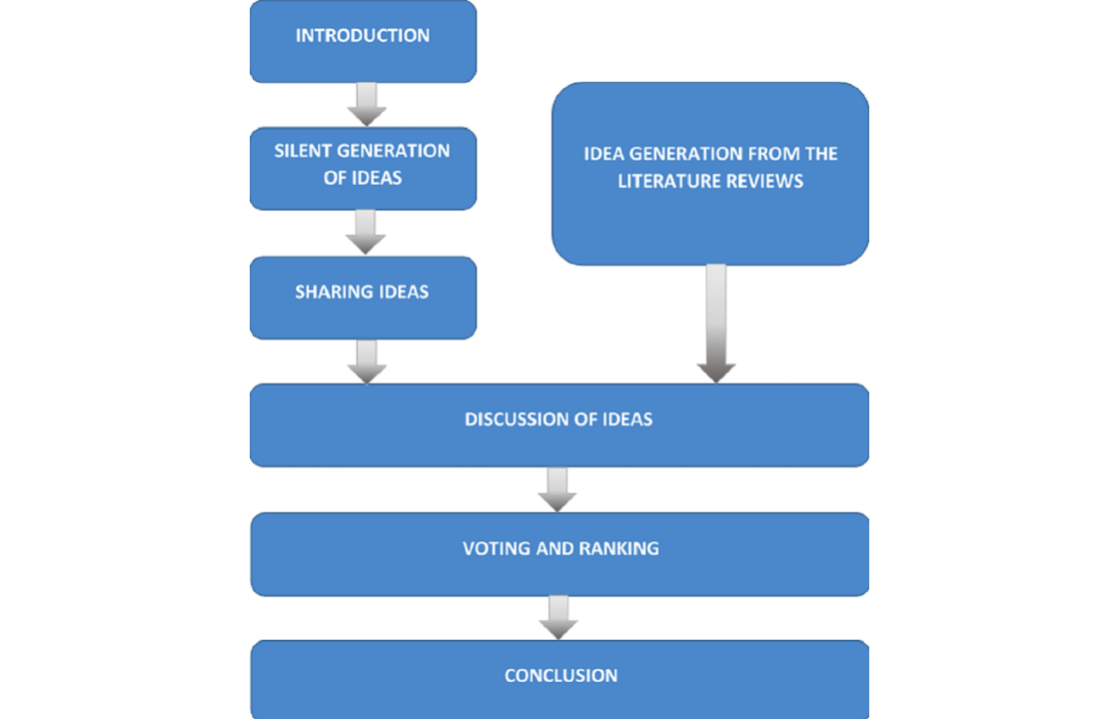
Flow of the process in the Nominal Group Technique.

The session was conducted in the following sequence (Figure 1):

1. Introduction to problem statement and explanation: Moderator briefed the participants on the flow of the session. They were reminded about the expected outcome from the discussion and emphasized on the importance of their contribution to the public benefit.
2. Silent generation of ideas in writing: Each participant was asked to write down their own ideas on what information on colorectal cancer that the community would like to know and what are the mobile app features that will make the apps user friendly and attractive to be used. This session does not allow any discussion and ended after 20 min.
3. Sharing ideas: The participants were invited to share their answers in the round robin manner. Each idea was written on the white board by the moderator. This session does not allow any discussion or argument of the ideas and ended after all participants had shared their answers.
4. Discussion of ideas: The participants were invited to give a verbal explanation about their answers that had been written on the board. They were asked to justify the need of the information and the prototype features to be included in the mobile app. Other participants were encouraged to actively discuss the relevancy of each point.
5. Voting and ranking: Participants were asked to vote their agreed answers based on the discussion. The votes will be tallied to produce the ranking of ideas from highest to lowest based on the research questions.
6. Conclusion: The session was concluded by the moderators. The agreed points were written on the white board.

### Prototype Development

The mobile app prototype development was conducted from December 2017 to March 2018. It was developed using the Ionic Framework that is based on the Apache Cordova and Angular JS. A total of 2 researchers who attended training of mobile app development had developed the prototype on the Android platform, which is the main platform for the majority of mobile phones in Malaysia [12]. The NGT findings were used to guide the content as well as features of the prototype. The prototype was then pretested using the same NGT participants for the comprehensibility and clarity as well as technical error. All feedback from the pretests were reviewed and addressed accordingly. The finalized prototype, named *ColorApp* (Colorectal Cancer Awareness Application), sized 9.5 MB was successfully uploaded in early March 2018 onto the Google Developer Console (Google Inc, Mountain View, CA, USA) and published in the Google Play store as a beta option for assessment purposes. It will be released free of charge as a production option for public use as part of the public health contribution once this study is completed.

### Prototype Usability Assessment

A cross-sectional study was conducted from February 2018 to March 2018 to assess the usability of the mobile app prototype. It was conducted along with the national-level community empowerment program, *Komuniti Sihat Pembina Negara* (KOSPEN; translates as *Healthy Community, Nation Builder*), in Kota Setar district in Kedah, which is in the northern state of Peninsular Malaysia. KOSPEN is one of the government initiatives for empowering the community for the prevention and control of non-communicable disease in Malaysia. There are a total of 153 localities in Kota Setar district, covering more than 20,000 people. This area was predetermined because of the distribution of KOSPEN localities in both urban and rural areas and good mobile broadband coverage.

Sample size was calculated using Gpower 3.1.9.2 for mean difference from a constant (1 sample case). The null hypothesis (H_0_) was the mean usability score of *ColorApp* is equal to 68 [13] and the alternative hypothesis was 73 (5 unit difference to H_0_). These hypotheses were analyzed using the One-sample *t* test. The alpha error was set at 5% and power was set at 80%. The estimated sample size was 33 after considering the detectable difference of 5 units. After an additional 25% anticipated community-based research dropout, the required sample size was 46. As 5 KOSPEN areas were selected, the sample size was rounded to 50. A total of 5 KOSPEN localities were randomly selected. In total, 10 participants aged 50 years and above who use mobile phone with the Android platform and had never been diagnosed with any type of cancer were randomly selected from each locality based on a list provided by the KOSPEN volunteer in the locality to be included in this study. All eligible participants were invited to attend 2 meeting sessions at 2 weeks interval. The first session introduced them on the mobile app. The participants were asked to download and install the mobile app from the Google Play store. They were required to use and interact with the mobile app at their own convenient time within the next 2 weeks. The second session was held after 2 weeks, where all participants were required to answer a validated questionnaire to assess the usability of the mobile app.

### Measuring Tool

The usability of the mobile app prototype was assessed using the validated Malay version of *System Usability Scale Questionnaire for the Assessment of Mobile Apps* (in Malay language, it is known as *Skala Kebolehgunaan Aplikasi Mudah Alih*). It is a 10-item questionnaire translated from the original English version, which is the system usability scale (SUS) questionnaire, into local Malay language. This Malay version had been validated to assess the usability of a mobile app [14]. The response score is calculated using the 5-point Likert scale ranging from 1 (strongly disagree) to 5 (strongly agree). The overall score is computed as the summation of all item scores multiplied by 2.5 and can range from 0 to 100. A standard usability score value of 68 was recommended by the original author to indicate good usability of an app [15]. The Cronbach alpha is 0.85. For the purpose of assessment of this mobile app, it was constructed as an online questionnaire using the Google form and the URL link was embedded in the mobile app prototype. Data entry and statistical analysis were made using IBM SPSS version 24.0. The 1-sample *t* test was used to determine whether the mean (SD) usability score of this mobile app is significantly higher than the standard usability score value of 68, with a level of significance (alpha error) less than .05.

This study has been approved by the National Medical Research Registry, Malaysia (NMRR-17-2623-38675 [IIR]), and Human Research Ethics Committee USM, Malaysia (USM/JEPeM/17110601).

## Results

### Nominal Group Technique Outputs

All participants have agreed that the mobile app prototype must cover the following topics:

1. Introduction to colorectal cancer
2. Sign and symptoms
3. Risk factors
4. Prevention
5. Colorectal cancer screening program

Malay language was chosen to be the main language as it is the national language, with consideration to add other languages in the future. Colorectal cancer was agreed to be translated into the Malay language as *Kanser Kolorektal* instead of the general term of bowel cancer, which is *Kanser Usus* in Malay. This terminology of *Kanser Kolorektal* was agreed to be used to introduce this disease to the community as it is more precise in referring to the disease. The introduction on colorectal was agreed to be of importance to introduce the terminology. The intended user group representative had agreed that the terminology was acceptable and understandable to the community after reading the introduction section.

The features of the mobile app prototype that were agreed upon are simple to operate, using easily understandable language, point-form information, infographic design, include a video, and interactive.

### Mobile App Prototype

**Figure 2:**
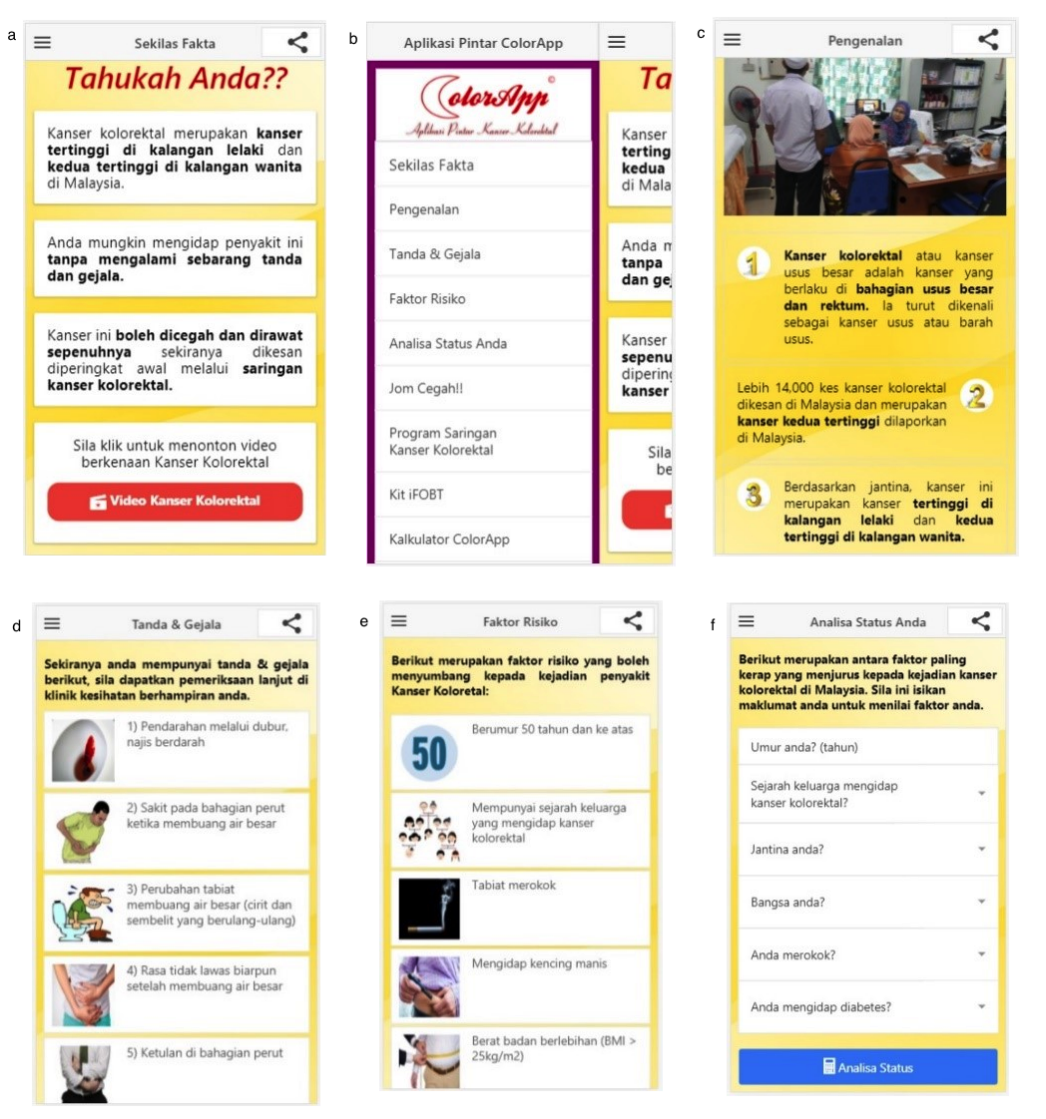
Screenshots of the main content of ColorApp: (a) "Do You Know" page; (b); drop-down menu (c) "Introduction" page; (d) "Sign & Symptoms" page; (e) "Risk Factors" page; (f) "Analyse Your Status" page.

**Figure 3:**
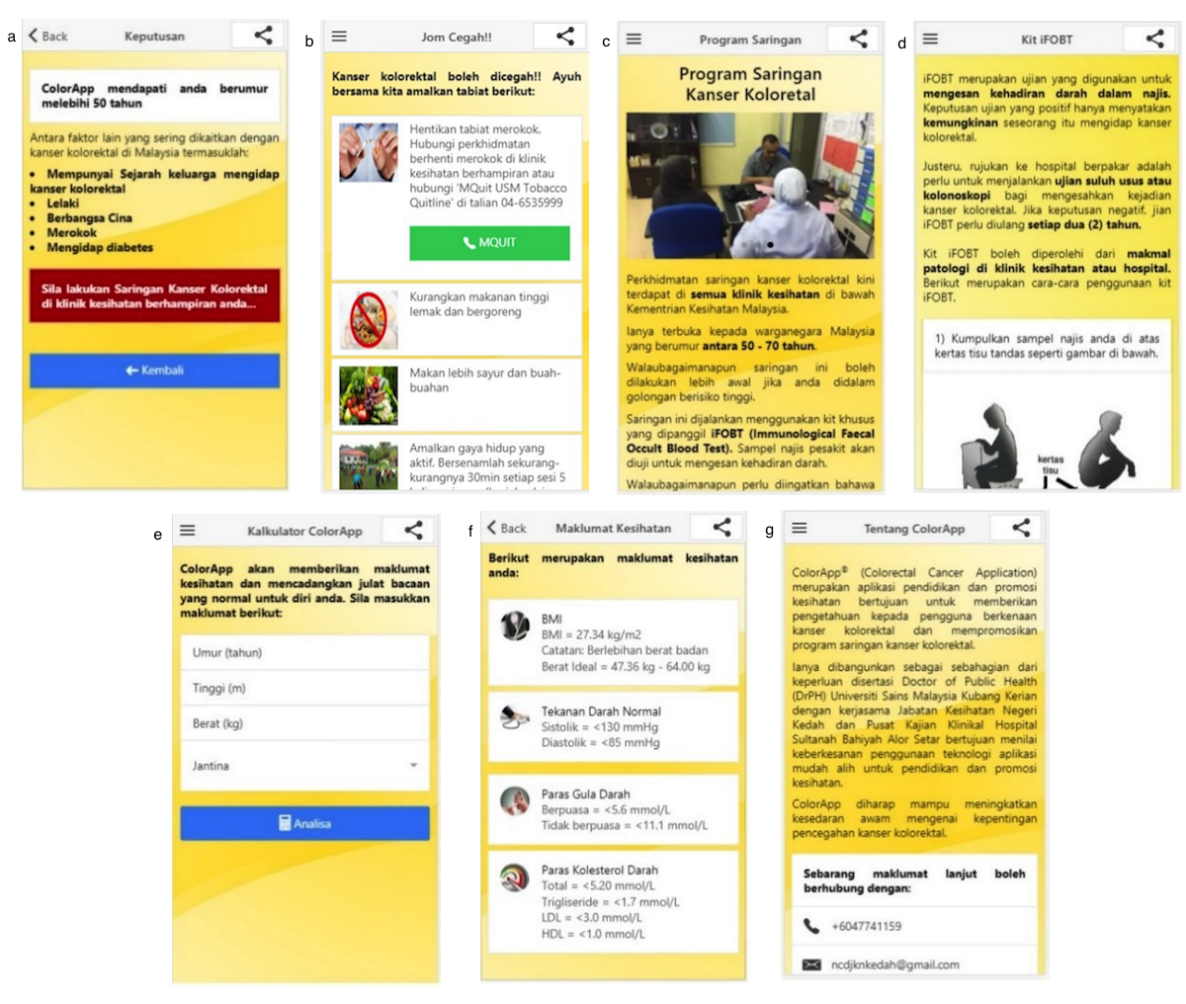
Screenshots of the main content of ColorApp: (a) "Result" page; (b) "Prevention of Colorectal Cancer" page; (c) "Screening Program" page; (d) "iFOBT Kit" page; (e) "ColorApp Calculator" page; (f) "Health Information" page; (g) "About ColorApp" page. iFOBT: immunochemical fecal occult blood test.

The mobile app prototype was developed for the Android platform. It is called *ColorApp*, the abbreviation of Colorectal Cancer Awareness Application. Figures 2 and 3 show the screenshots of the main content of *ColorApp*.

Section *Do You Know* (Figure 2, panel a) is the first section that will be seen by the user when they open *ColorApp*. It highlighted the important facts on colorectal cancer that the user needs to know. The user also can watch a short video on colorectal cancer. This is the only section wherein the user is required to have internet access. Other sections can be accessed using a drop-down menu (Figure 2, panel b). The user can click any of the menu options to go to the desired section. Figure 2, panel c, shows the section *Introduction*. The user will be introduced to colorectal cancer. Information was delivered in a point form to ease user’s reading and understanding. Pictures of colorectal cancer can be accessed by swiping to the right or left. The section *Sign & Symptoms* listed signs and symptoms of colorectal cancer that can be identified by a layman in a pictorial form (Figure 2, panel d). The user can learn about the risk factors of colorectal cancer through the section *Risk Factor* (Figure 2, panel e). Section *Analyse Your Status* is part of the artificial intelligence features of *ColorApp* that enable the user to enter their particulars, such as age, family history, sex, race, smoking status, and diabetes status (Figure 2, panel f). Once the user clicks on the button *Analyse Status*, *ColorApp* will assess user risk and advise the user on the need for colorectal cancer screening (Figure 3, panel a). Figure 3, panel b, shows the prevention of colorectal cancer. This section enables the user to directly call MQuit Tobacco Quitline for stop smoking service. Screening for colorectal cancer is introduced to the user via the section *Screening Program* (Figure 3, panel c). This section gives information on the criteria for screening, when and where the screening is available. This section is followed by *immunochemical fecal occult blood test kit* step-by-step instructions (Figure 3, panel d). The section *ColorApp Calculator* is another interactive section whereby the user can enter their age, height, weight, and sex to calculate their body mass index and ideal body weight (Figure 3, panels e and f). This section also provides the recommended level for blood pressure, sugar, and cholesterol. The last section is *About ColorApp* (Figure 3, panel g). This section gives information about this mobile app, email, and contact number for further information. The user also can share this mobile app via social media such as Facebook, WhatsApp, and Twitter with their family and friends.

### Usability

In total, 50 participants were involved in the usability assessment of *ColorApp*. Table 1 shows the sociodemographic characteristics of the participants in this study.

The usability score for *ColorApp* prototype usability showed a mean score difference of 4.9 (*P*=.004; 95% CI 1.626-8.174), totalling 72.9 (SD 11.52), higher than 68 on the Skala Kebolehgunaan Aplikasi Mudah Alih score which is the minimum cutoff point for a usable system. This indicates good usability of *ColorApp* as a mobile app, and, hence, a usable tool for colorectal cancer community education.

**Table 1:** Sociodemographic characteristics of participants (N=50).

Characteristics		Statistics
Age (years), mean (SD)		56.0 (5.69)
**Sex, n (%)**		
	Male	25 (50)
	Female	25 (50)
**Education, n (%)**		
	Primary	10 (20)
	Secondary	33 (66)
	Tertiary	7 (14)
**Occupation, n (%)**		
	Unemployed	2 (4)
	Self-employed	12 (24)
	Retired	1 (2)
	Clerical work	32 (64)
	Professional	3 (6)

## Discussion

### Principal Findings

The theory of HBM, which was adopted to develop the content of *ColorApp* prototype, addresses the perceived susceptibility, benefit, and health-seeking behavior of the user. According to HBM, the improvement of health-seeking behavior toward colorectal cancer prevention is related to the perception of an individual that he/she is susceptible to suffer from colorectal cancer, severity of colorectal cancer, and the benefit as well as barrier of preventive action including performing the colorectal cancer screening at the health clinic [16]. All these elements were addressed by the information in textual information, graphic presentation, and video. The interactive page analyzes the user’s risk for colorectal cancer and provides advice of action. This will further enhance the perceived susceptibility that the user might have the risk of getting the disease. Combination of various methods of knowledge dissemination will facilitate the knowledge transfer to the user.

The content of *ColorApp* was developed through NGT. NGT is a simplified semiqualitative method to look into preferences of the intended group [17]. NGT allows everyone in the group to contribute to the discussion, and with a good moderator, the likelihood of 1 person dominating the group process can be avoided. NGT also allows identification of important issues and prioritization for content development. Development of health education materials is very challenging, especially when the intended population is from outside the medical field [18]. The community has different levels of health literacy and might face difficulties in understanding certain terms or sentences, especially the health-related terminologies. Thus, the terminology of colorectal was discussed in great detail so as to decide whether to generalize it as bowel cancer or use the specific term *colorectal*. As the introduction had included the simple explanation of what is colorectal, the term colorectal was agreed to be used. Limited health literacy can limit users’ understanding, and in a worse scenario, it can pose a risk to them from misunderstanding [19]. The comprehensibility and consideration of health literacy had been addressed well with the involvement of representatives from intended users to ensure that the content of *ColorApp* is delivered clearly, particularly the actionable messages.

People aged 50 years and above are those who are at risk of getting colorectal cancer. This mobile app prototype, thus, is aimed to be used mainly by this group of people. Therefore, several issues that might be faced by the elderly when they use it must be considered. This included issues such as minimalist design to prevent cognitive overload in elderly population, large icons size that is easy to interpret for function and interaction logic, and simple navigation structure (such as back, forward buttons, or menu buttons) [20]. Although the main intended group includes people aged 50 years and above, this mobile app was designed to also be able to attract younger group of users through the combination of different media such as images and video as well as the ability of it being shared via social media with their family and friends, as shown during the NGT [21].

The *ColorApp* was developed as a tool for community education for colorectal cancer prevention. Thus, the usability of the prototype is therefore playing an important role in determining its effectiveness to improve health knowledge and awareness of the user. Usability is defined as the extent to which a product can be used by intended users to achieve specific goals effectively and efficiently as well as providing user satisfaction in a specified context of use [22]. The usability score, which is higher than the minimum acceptable score for a usable mobile app, indicates that *ColorApp* is user friendly for the intended user.

### Strength and Limitation

The development of *ColorApp* for community education on colorectal cancer will become the new method in disseminating the information about diseases using a mobile app. The cost of developing it is relatively cheaper, compared with producing printed materials. It is also easily updated when there are changes in information or management protocols. The features of *ColorApp* that are sharable through social media will help in disseminating this mobile app in the community. Furthermore, the usage of mobile apps is the current trend in information search that should be used to educate the public and improve their knowledge. This app had been registered under the intellectual property of Universiti Sains Malaysia with co-ownership with the Ministry of Health Malaysia and is already available in the Google Play store for all Malay speaking communities that use Android. The term and condition of the Google Play store will be applied with the use of this app. The limitation of this study is that the mobile app prototype is only being developed for the Android platform because of time and logistic reason.

### Future Recommendation

In the future, *ColorApp* should be available in other platforms also, especially iOS. The language option also should be made available in English, Chinese, and Tamil as these languages are also used frequently in multiethnic communities in Malaysia. The use of various languages will benefit more users, especially those who prefer certain languages. This will enhance knowledge transfer and improve the user’s understanding. Future research also should look into the effectiveness of mobile apps usage in improving the knowledge on colorectal cancer and the attitude and practice toward prevention of this disease including the screening uptake.

### Conclusions

In conclusion, the newly developed *ColorApp*, a mobile app for community education on colorectal cancer prevention, which had been developed through NGT and usability process, is a potentially useful tool in the modern technology era. It is available in the Google Play store for free and can be shared and distributed among the community. This study will be extended with the collaboration of the Ministry of Health Malaysia and Clinical Research Center, Hospital Sultanah Bahiyah, Alor Setar, Kedah, to assess the effectiveness of *ColorApp* as a community education and promotion tool in improving user knowledge on colorectal cancer and attitude toward colorectal cancer screening program.

## Abbreviations

ColorApp: Colorectal Cancer Awareness Application
HBM: Health Belief Model
H_0_: null hypothesis
KOSPEN: Komuniti Sihat Pembina Negara
NGT: nominal group technique
SUS: system usability scale
